# Bromide Treatment

**Published:** 1907-07

**Authors:** 


### BROMIDE TREATMENT.

No form of bromide treatment will prove successful unless the very purest salts are employed.

The combination of the five bromides in Peacock's Bromides will give the best possible bromide results, simply because the salts employed in its manufacture are extraordinarily pure. They are made especially for the product and salt of their high purity can not be purchased in the open market. It is, therefore, no wonder why Peacock's Bromides has been so generally endorsed and particularly by neurologists; large users of bromides.

